# Trends in Mortality Related to Lung Cancer and Secondary Brain Metastasis Among Older Adults in the United States From 1999 to 2020; Insights From CDC‐WONDER

**DOI:** 10.1002/brb3.71377

**Published:** 2026-03-31

**Authors:** Muhammad Haleem Nasar, Eeshal Fatima, Abdul Ahad, Sehar Ul Duaa, Wania Sultan, Kumail Mustafa Ali, Asad Ali Ahmed Cheema, Malik W. Z. Khan, Syed Hashim Ali Inam, Irfan Ullah, Paul Ferguson

**Affiliations:** ^1^ Department of Medicine Northwest School of Medicine Peshawar Pakistan; ^2^ Department of Medicine Services Institute of Medical Sciences Lahore Pakistan; ^3^ Department of Medicine Naseer Teaching Hospital Peshawar Pakistan; ^4^ Department of Medicine Dow Medical College Dow University of Health Sciences Karachi Pakistan; ^5^ Department of Medicine Jinnah Sindh Medical University Karachi Pakistan; ^6^ Department of Medicine International School of Medicine International University of Kyrgyzstan Bishkek Kyrgyzstan; ^7^ Yale University School of Medicine New Haven Connecticut USA; ^8^ Department of Neurology Marshall University Neuroscience Huntington West Virginia USA; ^9^ Department of Internal Medicine Khyber Teaching Hospital Peshawar Pakistan; ^10^ Institute of Public Health and Social Sciences Khyber Medical University Peshawar Pakistan

**Keywords:** CDC WONDER, lung cancer, mortality, older adults, secondary brain metastasis

## Abstract

**Introduction:**

Lung cancer (LC) is a major cause of secondary brain metastasis (SBM); however, mortality patterns associated with these conditions among older adults in the United States remain understudied. This study aimed to investigate trends in age‐adjusted mortality rates (AAMRs) and identify demographic and geographical disparities from 1999 to 2020.

**Methods:**

This retrospective cross‐sectional analysis used death certificate data from the US Centers for Disease Control and Prevention Wide‐Ranging Online Data for Epidemiologic Research (CDC WONDER) database, spanning 1999–2020. The study focused on older adults (≥ 65 years), identifying cases where LC and SBM were recorded as either the underlying or contributing causes of death. AAMRs per 100,000 people, and annual percent changes (APCs) were calculated. Trends were analyzed by year, sex, race/ethnicity, and geographical region.

**Results:**

The overall AAMR dropped between 1999 and 2013, increased steadily until 2017 (APC:3.4; 95% CI: −1.25 to 5.4), and then stabilized until 2020. Men had higher AAMRs (14.3) compared to women (10.0). Among racial/ethnic groups, non‐Hispanic (NH) Whites (12.7) and NH Blacks (11.7) had the highest AAMRs. Most patients died at home (44.5%). Geographically, the Midwest (13.0) and rural areas (11.4) showed higher mortality, highlighting notable disparities. States with LC‐ and SBM‐related mortality rates in the 90th percentile included Arkansas, Mississippi, Vermont, Indiana, and West Virginia.

**Conclusion:**

Following an initial decline, mortality has risen again in recent years. Considerable disparities were noted among various demographics and regions. Equitable efforts, including early diagnosis and management, are essential to reduce the burden on high‐risk populations.

## Graphical abstract

Using CDC WONDER (1999–2020), we quantified 109,832 deaths with lung cancer and secondary brain metastasis among US adults ≥ 65 (overall AAMR 11.9/100,000). Mortality was highest in males, non‐Hispanic White and Black populations, rural residents, and the Midwest, with Arkansas, Mississippi, Vermont, Indiana, and West Virginia in the top 90th percentile.

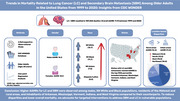

## 1 Introduction

Lung cancer (LC) is the most widespread cancer worldwide, representing 2.6 million cases, and contributing to 1 in 5 cancer‐related deaths (American Cancer Society 2024). In the United States (US), a new LC diagnosis occurs every 2 min, with over 356 daily deaths. (American Lung Association 2023). The 5‐year survival rate for LC is notably low, largely due to diagnosis at an advanced stage (American Lung Association 2023). LC predominantly affects individuals aged 65 and older, with an average diagnosis age of 70 (American Cancer Society 2026). In addition, bronchus and LC rank among the highest in economic cost globally, contributing significantly to 20.8% of the global economic cancer burden in the US (S. Chen et al. 2023). Despite projected declines in LC mortality by 2065, a substantial burden will persist, with an estimated 4.4 million LC deaths projected to occur in the US between 2015 and 2065 (Jeon et al. 2018).

Advancements in LC treatment over the past two decades have improved overall survival. However, prolonged survival has also been associated with a higher incidence of secondary brain metastasis (SBM), as patients live long enough to develop metastatic disease (Kratzer et al. 2024). Brain metastases are the most common intracranial neoplasm, affecting approximately 8%–10% of all cancer patients, and are frequently observed in those with LC (Kratzer et al. 2024; Goldberg et al. 2015). Among patients with LC, it is estimated that 10%–25% have brain metastases at diagnosis, and up to 40% develop intracranial metastases over the course of their disease (P. M. Chen et al. 2026). Despite therapeutic advances, LC‐associated SBM continues to carry a poor prognosis, with median survival as low as 12 months and 5‐year survival rates below 5% in the US (Sperduto et al. 2020; Shim et al. 2022). Moreover, patients with SBM experience longer hospital stays, more outpatient visits (Shim et al. 2022), increased healthcare costs (Shim et al. 2022; Fernandes et al. 2017), and a low quality of life (Peters et al. 2016; Naresh et al. 2021).

Despite the clinical significance of SBM in LC, mortality patterns specifically associated with concurrent LC and SBM have not been comprehensively explored in the US. Given the substantial morbidity, healthcare burden, and evolving therapeutic landscape, a detailed evaluation of demographic and geographic mortality trends is warranted. Therefore, this study aims to examine mortality patterns related to LC with SBM among older adults in the US.

## 2 Materials and Methods

### 2.1 STROBE Checklist

This study adhered to the Strengthening the Reporting of Observational Studies in Epidemiology (STROBE) guidelines (Cuschieri 2019) (Table S1).

### 2.2 Study Setting and Population

This retrospective cross‐sectional analysis used data for death certificates present in the Centers for Disease Control and Prevention Wide‐Ranging Online Data for Epidemiologic Research (CDC WONDER) database. A descriptive analysis of death rates among individuals with SBM and LC from 1999 to 2020 was performed. This database provides death certificate data from every US state and the District of Columbia. ICD‐10 (International Statistical Classification of Diseases and Related Health Problems, 10th Revision) codes C34 and C79.3 were used to identify deaths associated with LC and SBM, respectively, similar to previous studies (Crooks et al. 2024, Raisi‐Estabragh et al. 2022). Mortality data were analyzed using both the Underlying Cause of Death (UCD) and contributing cause of death, where UCD is defined by WHO as “the disease or injury that initiated the train of morbid events leading directly to death, or the circumstances of the accident or violence which produced the fatal injury,” whereas contributing causes include significant conditions that unfavorably influenced the course of the morbid process but were not the initiating cause (Centers for Disease Control and Prevention 2025). Death certificate information was obtained from the Multiple Cause of Death Public Use dataset (Centers for Disease Control and Prevention n.d.), and mortality among older individuals aged 65 years or above was explored. CDC WONDER is an anonymized government‐issued public‐use database; therefore, this study did not require institutional review board approval.

### 2.3 Data Extraction

Demographic variables studied included population count, gender, race/ethnicity, geographic location, urbanization, and location of death. The racial/ethnic categories were divided into Hispanic or Latino, non‐Hispanic (NH) White, NH Black/African American, NH American Indian/Alaska Native, and NH Asian/Pacific Islander. The Urban‐Rural Classification Scheme from the National Center for Health Statistics was used to categorize our study population by region. Metropolitan areas contained populations of 50,000 or more, while non‐metropolitan areas included locales with less than 50,000 residents. In addition, we stratified the US into four separate regions in line with the US Census Bureau's classification: Northeast, Midwest, South, and West (Ingram and Franco 2014).

### 2.4 Statistical Analysis

Sex, race, urbanization, and census‐based patterns were analyzed by determining crude and age‐adjusted mortality rates (AAMRs) per 100,000 individuals, standardized to the 2000 US population. This process applies the observed age‐specific death rates to a constant standard population, allowing a fair comparison of mortality risk over time and ensuring that any observed shifts in mortality reflect changes in disease burden or treatment outcomes rather than demographic shifts in the elderly population. Temporal shifts in death rates were assessed using the Joinpoint Regression Program (Version 5.0.2, National Cancer Institute) (National Cancer Institute n.d.), with log‐linear regression models used to calculate the annual percent change (APC) (Table S2) in AAMRs, along with their 95% confidence interval (CI). To determine the optimal number of joinpoints, we used the Weighted Bayesian Information Criterion (WBIC) as the model selection method, which provides more consistent results than traditional permutation tests in large population datasets (Kim et al. 2022). CIs for the APC were calculated using the t‐distribution method to ensure robust coverage across segmented trends (Kim et al. 2017). Deviation from the null hypothesis of zero change assessed whether APCs indicated an increasing or a decreasing trend. Statistical significance was obtained utilizing a two‐tailed *t*‐test with a cutoff of *p* < 0.05. Previous studies have used similar statistical analyses to analyze the data (Ahad et al. 2024; Shakil et al. 2024). In accordance with CDC WONDER data release standards, mortality rates were flagged as unreliable if based on fewer than 20 deaths. Such data points were excluded from the trend analysis to prevent statistical instability and to ensure the robustness of the reported APC (Centers for Disease Control and Prevention n.d.).

## 3 Results

Between 1999 and 2020, a cumulative total of 109,832 older individuals died with concurrent LC and SBM. Detailed records on the place of death were available for 103,641 cases. Of these, 44.5% died at home, 26.6% in medical facilities, 20.0% in nursing homes/long‐term care facilities, and 8.9% in hospices (Tables S3 and S4).

### 3.1 Annual Trend

**FIGURE 1:**
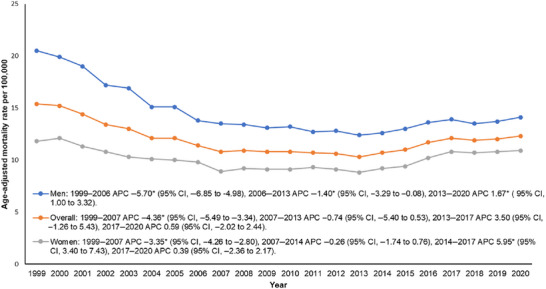
Overall and sex‐specific trends in age‐adjusted mortality rates associated with lung cancer and secondary brain metastasis among older adults in the United States from 1999 to 2020.

The overall AAMR decreased from 15.4 in 1999 to 12.3 in 2020. A significant decline was observed between 1999 and 2007, followed by a stable period between 2007 and 2013. Mortality then increased steadily until 2017 and remained stable through 2020 (Figure 1, Tables S4 and S5).

### 3.2 Demographic Variations in LC and SBM‐Related Mortality Trends

#### 3.2.1 Gender

Throughout the study period, men consistently had higher AAMRs (14.4) compared with women (10.0). For men, AAMR decreased significantly from 1999 to 2006, while for women it decreased from 1999 to 2007. Following this, AAMR for men continued to decrease modestly until 2013, whereas it remained stable for women until 2014. After 2013, AAMR significantly increased among men through 2020. In contrast, women experienced a sharp rise in mortality from 2014 to 2017, followed by stability through 2020 (Figure 1, Tables S2 and S5).

#### 3.2.2 Race

**FIGURE 2:**
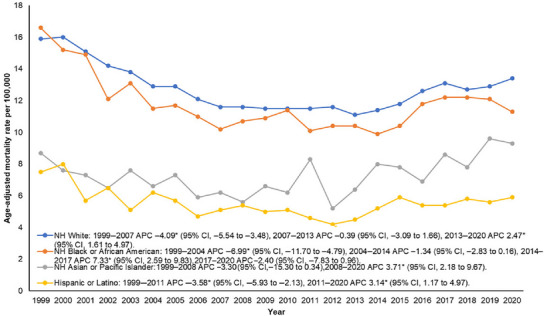
Race/Ethnicity‐specific trends in age‐adjusted mortality rates associated with lung cancer and secondary brain metastasis among older adults in the United States from 1999 to 2020.

Mortality was highest among NH Whites (AAMR: 12.7), followed by NH Blacks or African Americans (AAMR: 11.7), NH Asians or Pacific Islanders (AAMR: 7.5), and Hispanics or Latinos (AAMR: 5.5) (Table S6). Data for NH American Indian or Alaska Native individuals were unreliable and were excluded from the analysis. AAMR for NH Asians or Pacific Islanders decreased from 1999 to 2008, and for Hispanics or Latinos from 1999 to 2011. Thereafter, AAMRs increased in both groups until 2020. Among NH White individuals, AAMR decreased significantly between 1999 and 2007, while among NH Black or African American individuals mortality rate decreased between 1999 and 2004. Mortality rates remained stable until 2013 and 2014, respectively, for both groups. After this period, AAMR increased among NH Whites through 2020. Among NH Black individuals, AAMR increased until 2017 and then remained stable through 2020 (Figure 2, Tables S2 and S6).

#### 3.2.3 Geographic Variations in SBM and LC‐Related Mortality Trends

**FIGURE 3:**
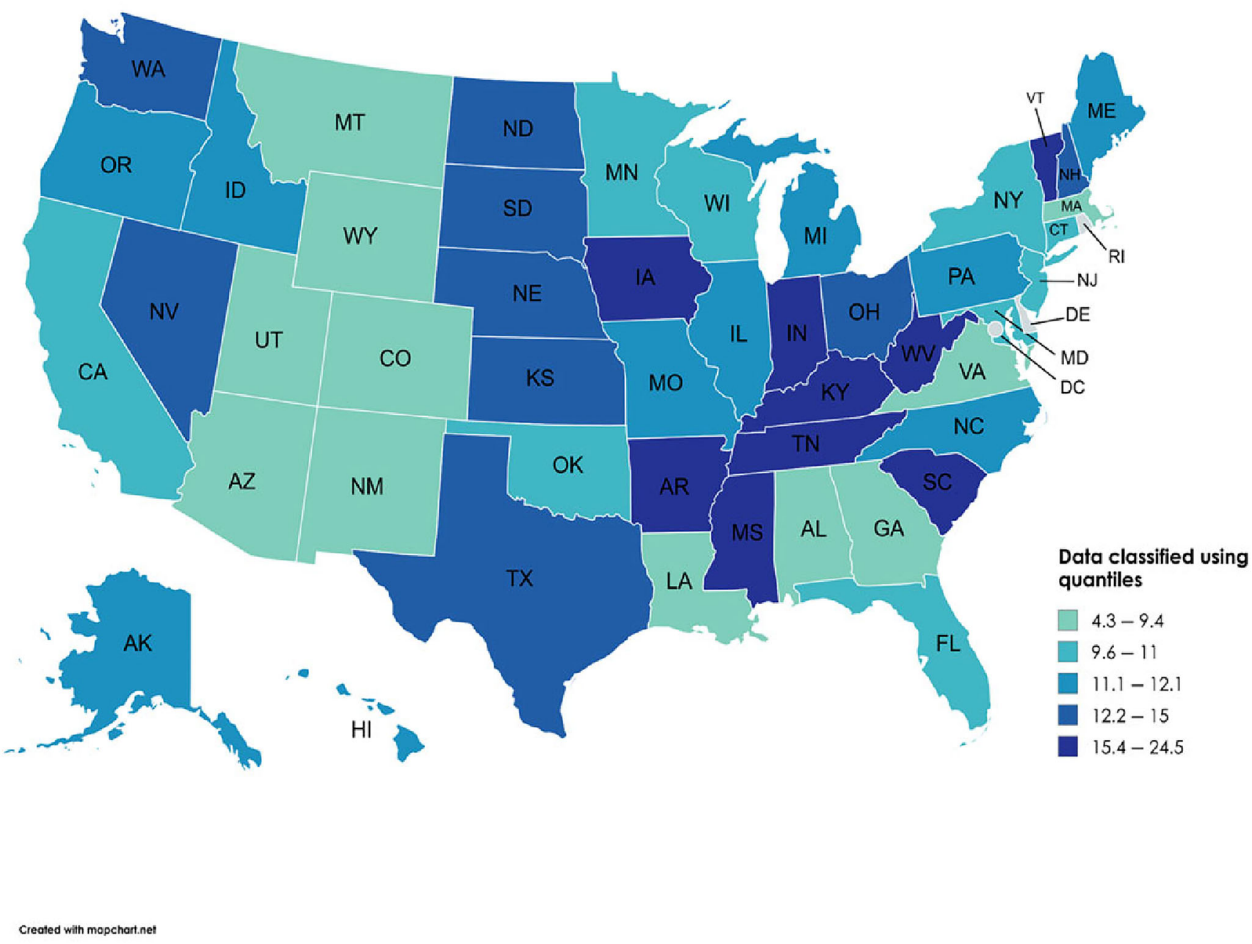
State‐level age‐adjusted mortality rates associated with lung cancer and secondary brain metastasis among older adults in the United States from 1999 to 2020.

AAMRs varied significantly across the states, ranging from as high as 24.5 in Arkansas to as low as 4.3 in Utah. (Figure 3, Table S7). States with AAMRs ranking in the upper 90th percentile included Arkansas, Mississippi, Vermont, Indiana, and West Virginia. These states exhibited almost triple the AAMRs compared to those in the lower 10th percentile, such as Louisiana, Montana, Arizona, the District of Columbia, and Utah.

Based on the census region, the Midwest region had the highest AAMR (13.0), followed by the South (12.5), West (10.8), and Northeast regions (10.5) (Table S8).

**FIGURE 4:**
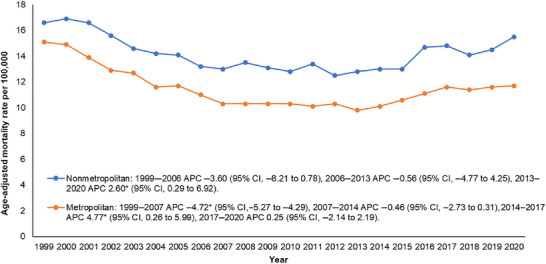
Urban‐rural classification‐specific trends in age‐adjusted mortality rates associated with lung cancer and secondary brain metastasis among older adults in the United States from 1999 to 2020.

By urbanization level, nonmetropolitan areas consistently had higher AAMRs (14.2) compared with metropolitan areas (11.4). AAMR decreased significantly between 1999 and 2007 in metropolitan areas and steadily in nonmetropolitan areas from 1999 to 2006. AAMR then remained stable until 2013 in nonmetropolitan areas and until 2014 in metropolitan areas. After 2013, metropolitan AAMR increased until 2017 and then remained stable. In contrast, nonmetropolitan AAMR increased from 2013 through 2020 (Figure 4, Tables S2 and S9).

### 3.3 Most Common Underlying Causes of Death

Among the 15 leading underlying causes of death in older adults in the US, diseases of the heart, malignant neoplasms, cerebrovascular diseases, chronic lower respiratory diseases, and Alzheimer's disease accounted for the highest number of deaths. A full list of causes and corresponding AAMR is provided in Table S10.

## 4 Discussion

We conducted the first retrospective analysis that studied the mortality trends of concomitant LC and SBM among older US adults using CDC WONDER. Initially, we observed a decreasing trend in overall mortality till 2013, followed by an increase till 2017, and finally a stable rate till 2020. Men had significantly higher AAMRs than women. NH Whites and Hispanics or Latinos had the highest and lowest mortality rates, respectively. Regional variations demonstrated that the Midwest region had the highest AAMRs. Among the states, Arkansas and Mississippi had the highest mortality rates, while the District of Columbia, and Utah had the lowest mortality rates. Our study also reported higher death rates in nonmetropolitan areas from 1999 to 2020.

Our study reported an overall decreasing trend of mortality in patients who have concomitant SBM and LC from 1999 to 2020. However, there have been various fluctuations in the trend, which include a rise from 2013 to 2017 and then a stable rate till 2020. The increase in the trend can be justified by a few reasons. First, the probability of death caused by cancer tends to increase in parallel with the progression of age (Weir et al. 2016). As the population of the US grows and gets older, the number of deaths from cancer also increases (Weir et al. 2016). Second, the occurrence of brain metastases is increasing in the US due to rising life expectancy (Kratzer et al. 2024). Third, the increase in the prevalence of smoking and pollution levels leads to an increase in LC burden (Sharma 2022). Meanwhile, the initially decreasing and later stabilizing trends can be attributed to advancements in the treatment of cancer over the past decades. Recently, newly developed drugs known as epidermal growth factor receptor (EGFR) tyrosine kinase inhibitors (TKIs), such as erlotinib, gefitinib, and afatinib, have demonstrated effectiveness in treating patients with brain metastases (D'Antonio et al. 2014). Administering EGFR TKIs results in a response rate of 70%–80% and leads to extended periods of progression‐free and overall survival compared to traditional chemotherapeutic regimens (D'Antonio et al. 2014). Another reason for initially declining trends could be the decrease in LC mortality since 1999 (Didier et al. 2024), likely due to improvements in early detection and therapeutic strategies. This trend may also be partly attributed to the substantial decline in smoking prevalence in the US from 1971 to 2020 (Kranjac and Kranjac 2025).

We also studied demographic variations in the mortality rates, and according to our analysis, men persistently recorded higher mortality rates thanwomen throughout the study duration. This is because, according to the studies conducted using the GLOBOCAN 2020 database and CDC WONDER, the incidence and mortality rates due to LC alone are higher in men than in women (Sharma 2022; Didier et al. 2024). Another retrospective study conducted in the US that studied the incidence of brain metastasis and the survival rate of patients with small cell lung cancer (SCLC) also reported that survival was higher in female patients (Sahmoun et al. 2005). A nationwide Flatiron electronic health record study also concluded that men have higher odds of developing brain metastases (Cioffi et al. 2024), which is consistent with our findings. Men are less likely to seek routine medical care or screening, which allows tumors to grow and progress to later stages before diagnosis (Cleary et al. 1982). Higher smoking rates among men might have also disproportionately affected the gender differences in the mortality trends (Centers for Disease Control and Prevention (CDC) 2012; Centers for Disease Control and Prevention (CDC) 2011).

The racial analysis reported the highest mortality in NH Whites and Blacks. Hispanics or Latinos showed the lowest mortality rates in our study, which was similar to another study that reported the epidemiological trends of LC (Houston et al. 2018). Another multicenter study conducted in the US studied the epidemiological trends of synchronous brain metastasis from 2010 to 2020 using Surveillance, Epidemiology, and End Results (SEER) database. This study reported that most participants were Whites, while the highest AAMR was observed in Whites and Blacks (Che et al. 2022), thus showing consistency with our results. The trends of synchronous brain metastasis from 2015 to 2019 were examined in another similar population‐based study conducted in the US using the SEER database, which also reported the highest incidence in NH Whites, followed by NH Blacks, which is again similar to our findings (Hayes et al. 2017). Furthermore, previous research has shown that African Americans are more likely to experience in‐hospital mortality, have SBM, and are less likely to participate in clinical trials or receive stereotactic radiosurgery (Hayes et al. 2017). These racial disparities can be explained by a number of factors, including environmental and lifestyle exposures (Houston et al. 2018). For instance, cigarette smoking, which is the main risk factor for LC, is more common in NH whites, while on the other hand, NH blacks are disproportionately employed in such jobs where they have more exposure to several pollutants, including diesel, asbestos, pesticides, and radon, which affect the air quality and hence, can explain the higher incidence and mortality due to LC in them (Houston et al. 2018). The lower mortality rates among Hispanics or Latinos can be explained by the Hispanic paradox, which describes the phenomenon whereby Hispanics or Latinos in the US often experience better health outcomes and longer life expectancy despite generally having lower socioeconomic status, including lower income and education levels, compared with NHs (Elsevier n.d.). Proposed explanations for this paradox include the healthy migrant effect, where healthier individuals are more likely to migrate, as well as potential inaccuracies in population enumeration that may contribute to discrepancies in health statistics (Elsevier n.d.). In addition, lower smoking prevalence rates in Hispanics or Latinos could be another reason (Espinosa et al. 2026). Socioeconomic status and access to healthcare resources are also major contributing factors to racial disparities (Parker et al. 2023). However, these differences need to be studied and addressed to ensure equitable healthcare provision across all racial subgroups.

In addition to demographics, we also studied geographic differences in the mortality trends. Midwest and South regions showed the highest mortality. This could be because mortality due to LC alone is higher in these regions, which can be attributed to the baseline population health and increased prevalence of smoking in these regions as compared to the Northeast and West (Didier et al. 2024). In addition, rural areas exhibited higher AAMRs than urban areas throughout the study period. A study that reported disparities in LC incidence rates concluded that the degree of disparity in the trends increased with increasing rurality of residence (Houston et al. 2018). The 10‐year incidence and mortality trends analysis by Che et al. of LC and synchronous brain metastasis in the US also reported considerably higher mortality in rural areas (Che et al. 2022). This could be attributed to lower household income, lower socioeconomic status, lack of adequate healthcare resources, and less physician care in rural areas in comparison to urban areas (Didier et al. 2024; Che et al. 2022; Basu et al. 2019). Other potential contributing factors to rural‐urban disparities could be educational status, screening programs, and timing of diagnosis. Nevertheless, it is important to take these differences into account for a better understanding of trends across different regions.

Brain metastases and LC are major contributors to morbidity and mortality. It is important to closely monitor the risk factors responsible for individuals at higher risk. For instance, a substantial proportion of mortality occurs in rural areas. With approximately one‐fifth of the US population residing in rural areas, there is a clear need for targeted and innovative healthcare strategies. Similarly, the implementation of intensive smoking cessation programs, particularly in males, high‐risk regions, and states, can reduce the incidence of LC, thereby mitigating healthcare burden among high‐risk groups. In addition, the early detection of asymptomatic brain metastases in the vulnerable subpopulations can be treated more effectively with therapies, including targeted treatments, which can improve outcomes. Specific measures, such as ensuring equitable access to healthcare, personalized awareness programs, early diagnostic and risk‐stratified screening initiatives, can offer advantages to at‐risk subgroups. Furthermore, future research should focus on investigating the potential biological, socioeconomic, and healthcare system factors that contribute to these gender, racial, and regional disparities.

This analysis harbors certain limitations that should be considered. First, the use of ICD codes and reliance on death certificates increases the likelihood of incorrect diagnoses, misinterpretation, omission, and transcription errors in data, leading to a certain degree of inaccuracy. Second, CDC WONDER does not provide data pertaining to histological subtypes or other cancer‐related specifications. Third, although previous research has shown that death certificates are generally reliable for identifying LC mortality, a limitation is that we do not have similar studies validating death certificates specifically for SBM (Doria‐Rose and Marcus 2009; Marcus et al. 2016). Fourth, analyses at the level of US census regions may be affected by ecological bias, as CDC WONDER provides primarily aggregated population‐level data rather than individual‐level information, making it important to interpret group‐level associations with caution. Lastly, most of the data for NH American Indians in the racial analysis were unreliable and were therefore excluded from the joinpoint regression analysis.

## 5 Conclusion

In conclusion, after more than a decade‐long decline, LC and SBM mortality rates rose and then stabilized. Most pronounced mortality rates were seen in the male population, NH Whites, and the Midwest region. Equitable efforts, including early diagnosis and prompt treatment or management strategies, are required to reduce the burden on high‐risk populations.

## Author Contributions

All authors contributed to the study conception and design. Material preparation, data collection, and analysis were performed by Muhammad Haleem Nasar, Eeshal Fatima, Abdul Ahad, Sehar Ul Duaa, and Wania Sultan. The first draft of the manuscript was written by Muhammad Haleem Nasar, Eeshal Fatima, Abdul Ahad, Sehar Ul Duaa, Wania Sultan, Kumail Mustafa Ali, Asad Ali Ahmed Cheema, and Malik W.Z. Khan. Data analysis and methodology contributions were made by Muhammad Haleem Nasar, Eeshal Fatima, Syed Hashim Ali Inam, and Irfan Ullah. Muhammad Haleem Nasar, Kumail Mustafa Ali, Asad Ali Ahmed Cheema, Syed Hashim Ali Inam, Irfan Ullah, and Paul Ferguson contributed to reviewing and editing the manuscript. All authors read and approved the final manuscript.

## Funding

The authors have nothing to report.

## Ethics Statement

The authors have nothing to report.

## Conflicts of Interest

The authors declare no conflicts of interest.

## Supporting information




**Supplementary Table S1‐S10**: brb371377‐sup‐0001‐TableS1‐S10.docx

## Data Availability

All data utilized in this research were sourced from the CDC WONDER database. CDC WONDER is a publicly available government‐issued database, freely accessible online through https://wonder.cdc.gov/mcd‐icd10.html.
