# Supplementary material for: Trends in Mortality Related to Lung Cancer and Secondary Brain Metastasis Among Older Adults in the United States From 1999 to 2020; Insights From CDC‐WONDER
Source: Brain Behav. 2026 Mar 31;16(4):e71377. doi: 10.1002/brb3.71377 (PMC13111998; doi:10.1002/brb3.71377)
Supplement: Supplementary file 1 — Supplementary Table S1‐S10: brb371377‐sup‐0001‐TableS1‐S10.docx [file BRB3-16-e71377-s001.docx]

**Supplementary Tables**

**Supplemental Table 1**: STROBE checklist showing adherence to reporting guidelines for this cross-sectional study.

STROBE Statement—Checklist of items that should be included in reports of ***cross-sectional studies***

|  | Item No | Recommendation | Page  Number | Relevant text from the manuscript |
| --- | --- | --- | --- | --- |
| **Title and abstract** | 1 | (*a*) Indicate the study’s design with a commonly used term in the title or the abstract | 4 | Abstract Paragraph 2 |
|  |  | (*b*) Provide in the abstract an informative and balanced summary of what was done and what was found | 4 | Abstract Paragraph 2 |
| Introduction | | |  |  |
| Background/rationale | 2 | Explain the scientific background and rationale for the investigation being reported | 5 | Introduction Paragraph 1 and 2 |
| Objectives | 3 | State specific objectives, including any prespecified hypotheses | 5 | Introduction Paragraph 3 |
| Methods | | |  |  |
| Study design | 4 | Present key elements of study design early in the paper | 5 | Methods Paragraph 2 |
| Setting | 5 | Describe the setting, locations, and relevant dates, including periods of recruitment, exposure, follow-up, and data collection | 5 | Methods Paragraph 2 |
| Participants | 6 | (*a*) Give the eligibility criteria, and the sources and methods of selection of participants | 5 | Methods Paragraph 2 |
| Variables | 7 | Clearly define all outcomes, exposures, predictors, potential confounders, and effect modifiers. Give diagnostic criteria, if applicable |  | NA |
| Data sources/ measurement | 8* | For each variable of interest, give sources of data and details of methods of assessment (measurement). Describe comparability of assessment methods if there is more than one group | *5* | Methods Paragraph 2 |
| Bias | 9 | Describe any efforts to address potential sources of bias |  | NA |
| Study size | 10 | Explain how the study size was arrived at | 6 | Methods Paragraph 4 |
| Quantitative variables | 11 | Explain how quantitative variables were handled in the analyses. If applicable, describe which groupings were chosen and why | 5-6 | Methods Paragraph 3 |
| Statistical methods | 12 | (*a*) Describe all statistical methods, including those used to control for confounding | 6 | Methods Paragraph 4 |
|  |  | (*b*) Describe any methods used to examine subgroups and interactions | NA | NA |
|  |  | (*c*) Explain how missing data were addressed | 6 | Methods Paragraph 3 |
|  |  | (*d*) If applicable, describe analytical methods taking account of sampling strategy | NA | NA |
|  |  | (*e*) Describe any sensitivity analyses | NA | NA |
| Results | | |  |  |
| Participants | 13* | (a) Report numbers of individuals at each stage of study—eg numbers potentially eligible, examined for eligibility, confirmed eligible, included in the study, completing follow-up, and analysed | 6 | Results Paragraph 1 |
|  |  | (b) Give reasons for non-participation at each stage |  | NA |
|  |  | (c) Consider use of a flow diagram |  | NA |
| Descriptive data | 14* | (a) Give characteristics of study participants (eg demographic, clinical, social) and information on exposures and potential confounders | 5-6 | Results Paragraph 3-7 |
|  |  | (b) Indicate number of participants with missing data for each variable of interest |  | NA |
| Outcome data | 15* | Report numbers of outcome events or summary measures |  | NA |
| Main results | 16 | (*a*) Give unadjusted estimates and, if applicable, confounder-adjusted estimates and their precision (eg, 95% confidence interval). Make clear which confounders were adjusted for and why they were included | 5-6 | Results Paragraph 2-7 |
|  |  | (*b*) Report category boundaries when continuous variables were categorized |  | NA |
|  |  | (*c*) If relevant, consider translating estimates of relative risk into absolute risk for a meaningful time period |  | NA |
| Other analyses | 17 | Report other analyses done—eg analyses of subgroups and interactions, and sensitivity analyses |  | NA |
| Discussion | | |  |  |
| Key results | 18 | Summarise key results with reference to study objectives | 7-9 | Discussion Paragraph 1-6 |
| Limitations | 19 | Discuss limitations of the study, taking into account sources of potential bias or imprecision. Discuss both direction and magnitude of any potential bias | 9 | Discussion Paragraph 7 |
| Interpretation | 20 | Give a cautious overall interpretation of results considering objectives, limitations, multiplicity of analyses, results from similar studies, and other relevant evidence | 9 | Discussion Paragraph 7 |
| Generalisability | 21 | Discuss the generalisability (external validity) of the study results | 9 | Discussion Paragraph 6 |
| Other information | | |  |  |
| Funding | 22 | Give the source of funding and the role of the funders for the present study and, if applicable, for the original study on which the present article is based | 10 | NA |

*Give information separately for exposed and unexposed groups.

NA: Not applicable

**Note:** An Explanation and Elaboration article discusses each checklist item and gives methodological background and published examples of transparent reporting. The STROBE checklist is best used in conjunction with this article (freely available on the Web sites of PLoS Medicine at http://www.plosmedicine.org/, Annals of Internal Medicine at http://www.annals.org/, and Epidemiology at http://www.epidem.com/). Information on the STROBE Initiative is available at www.strobe-statement.org.

**Supplemental Table 2:** Annual percent change (APC) of lung cancer and secondary brain metastasis–related age-adjusted mortality rates per 100,000 among older adults in the United States, 1999 to 2020.

| **Year Interval** | **APC (95% CI)** |
| --- | --- |
| **Overall** | |
| 1999-2007 | -4.36* (-5.49 to -3.34) |
| 2007-2013 | -0.74 (-5.40 to 0.53) |
| 2013-2017 | 3.50 (-1.26 to 5.43) |
| 2017-2020 | 0.59 (-2.02 to 2.44) |
| **Men** | |
| 1999-2006 | -5.70* (-6.85 to -4.98) |
| 2006-2013 | -1.40* (-3.29 to -0.08) |
| 2013-2020 | 1.67* (1.00 to 3.32) |
| **Women** | |
| 1999-2007 | -3.35* (-4.26 to -2.80) |
| 2007-2014 | -0.26 (-1.74 to 0.76) |
| 2014-2017 | 5.95* (3.40 to 7.43) |
| 2017-2020 | 0.39 (-2.36 to 2.17) |
| **NH White** | |
| 1999-2007 | -4.09* (-5.54 to -3.48) |
| 2007-2013 | -0.39 (-3.09 to 1.66) |
| 2013-2020 | 2.47* (1.61 to 4.97) |
| **NH Black or African American** | |
| 1999-2004 | -6.99* (-11.70 to -4.79) |
| 2004-2014 | -1.34 (-2.83 to 0.16) |
| 2014-2017 | 7.33* (2.59 to 9.83) |
| 2017-2020 | -2.40 (-7.83 to 0.96) |
| **Hispanic or Latino** | |
| 1999-2011 | -3.58* (-5.93 to -2.13) |
| 2011-2020 | 3.14* (1.17 to 4.97) |
| **NH Asian or Pacific Islander** | |
| 1999-2008 | -3.30 (-15.30 to 0.34) |
| 2008-2020 | 3.71* (2.18 to 9.67) |
| **Nonmetropolitan areas** | |
| 1999-2006 | -3.60 (-8.21 to 0.78) |
| 2006-2013 | -0.56 (-4.77 to 4.25) |
| 2013-2020 | 2.60* (0.29 to 6.92) |
| **Metropolitan area** | |
| 1999-2007 | -4.72* (-5.27 to -4.29) |
| 2007-2014 | -0.46 (-2.73 to 0.31) |
| 2014-2017 | 4.77* (0.26 to 5.99) |
| 2017-2020 | 0.25 (-2.14 to 2.19) |
| APC = annual percent change; NH = non-Hispanic; * Indicates that the annual percentage change (APC) is significantly different from zero at α = 0.05. AAMR = age-adjusted mortality rate. |  |

**Supplemental Table 3:** Lung cancer and secondary brain metastasis–related deaths, stratified by sex and race, among older adults in the United States, 1999 to 2020.

|  | | | | | | | | | |  |
| --- | --- | --- | --- | --- | --- | --- | --- | --- | --- | --- |
| **Year** | **Overall** | **Women** | **Men** | **NH White** | **NH Black or African American** | **NH Asian or Pacific Islander** | **Hispanic or Latino** | | |  |
| 1999 | 5364 | 2364 | 3000 | 4674 | 473 | 71 | 126 | | |  |
| 2000 | 5348 | 2435 | 2913 | 4684 | 437 | 67 | 138 | | |  |
| 2001 | 5084 | 2274 | 2810 | 4442 | 435 | 66 | 107 | | |  |
| 2002 | 4756 | 2183 | 2573 | 4180 | 359 | 65 | 125 | | |  |
| 2003 | 4669 | 2105 | 2564 | 4069 | 392 | 78 | 106 | | |  |
| 2004 | 4391 | 2084 | 2307 | 3816 | 351 | 71 | 130 | | |  |
| 2005 | 4427 | 2075 | 2352 | 3836 | 364 | 81 | 126 | | |  |
| 2006 | 4216 | 2044 | 2172 | 3661 | 352 | 71 | 111 | | |  |
| 2007 | 4073 | 1899 | 2174 | 3530 | 328 | 76 | 123 | | |  |
| 2008 | 4220 | 1998 | 2222 | 3638 | 354 | 74 | 138 | | |  |
| 2009 | 4255 | 2021 | 2234 | 3643 | 368 | 93 | 133 | | |  |
| 2010 | 4327 | 2043 | 2284 | 3677 | 396 | 89 | 140 | | |  |
| 2011 | 4406 | 2139 | 2267 | 3749 | 364 | 128 | 137 | | |  |
| 2012 | 4545 | 2158 | 2387 | 3921 | 386 | 85 | 131 | | |  |
| 2013 | 4576 | 2166 | 2410 | 3880 | 407 | 116 | 149 | | |  |
| 2014 | 4862 | 2324 | 2538 | 4094 | 410 | 148 | 180 | | |  |
| 2015 | 5217 | 2471 | 2746 | 4348 | 447 | 167 | 216 | | |  |
| 2016 | 5660 | 2740 | 2920 | 4726 | 530 | 150 | 210 | | |  |
| 2017 | 6095 | 2992 | 3103 | 5061 | 577 | 197 | 221 | | |  |
| 2018 | 6154 | 3058 | 3096 | 5070 | 592 | 190 | 253 | | |  |
| 2019 | 6451 | 3186 | 3265 | 5306 | 611 | 247 | 253 | | |  |
| 2020 | 6736 | 3301 | 3435 | 5591 | 586 | 249 | 275 | | |  |
| **Total** | 109832 | 52060 | 57772 | 93596 | 9519 | 2579 | 3528 | | |  |
| NH= non-Hispanic |  |  |  |  |  |  |  |  |  | |

**Supplemental Table 4:** Lung cancer and secondary brain metastasis–related mortality, stratified by place of death, among older adults in the United States, 1999 to 2020.

| **Deaths** | | | | |  |
| --- | --- | --- | --- | --- | --- |
| **Year** | **Medical Facility** | **Home** | **Nursing Home/Long-term care Facility** | **Hospices** |  |
|  |  |  |  |  |  |
| 1999 | 1714 | 2057 | 1145 | Missing |  |
| 2000 | 1681 | 2110 | 1119 | Missing |  |
| 2001 | 1585 | 1930 | 1142 | Missing |  |
| 2002 | 1417 | 1825 | 1055 | Missing |  |
| 2003 | 1340 | 1886 | 959 | 32 |  |
| 2004 | 1195 | 1789 | 920 | 45 |  |
| 2005 | 1226 | 1745 | 915 | 131 |  |
| 2006 | 1088 | 1629 | 937 | 173 |  |
| 2007 | 1072 | 1631 | 842 | 211 |  |
| 2008 | 1100 | 1580 | 857 | 300 |  |
| 2009 | 1051 | 1669 | 831 | 328 |  |
| 2010 | 964 | 1787 | 849 | 388 |  |
| 2011 | 972 | 1812 | 885 | 419 |  |
| 2012 | 971 | 1826 | 863 | 532 |  |
| 2013 | 977 | 1953 | 821 | 440 |  |
| 2014 | 939 | 2082 | 870 | 650 |  |
| 2015 | 1055 | 2316 | 861 | 690 |  |
| 2016 | 1075 | 2443 | 926 | 883 |  |
| 2017 | 1157 | 2658 | 973 | 971 |  |
| 2018 | 1184 | 2662 | 942 | 966 |  |
| 2019 | 1194 | 2868 | 911 | 1046 |  |
| 2020 | 1076 | 3513 | 718 | 864 |  |
| **Total** | 26002 | 45771 | 20341 | 9069 |  |

**Supplemental Table 5:** Overall and sex‐stratified lung cancer and secondary brain metastasis–related age-adjusted mortality rates per 100,000 among older adults in the United States, 1999 to 2020.

| **Age-Adjusted Rate (95% CI)** | | | |
| --- | --- | --- | --- |
| **Year** | **Men** | **Women** | **Overall** |
| 1999 | 20.5 (19.8-21.3) | 11.8 (11.3-12.2) | 15.4 (15.0-15.8) |
| 2000 | 19.9 (19.2-20.6) | 12.1 (11.6-12.5) | 15.2 (14.8-15.7) |
| 2001 | 19.0 (18.3-19.7) | 11.3 (10.8-11.7) | 14.4 (14.0-14.8) |
| 2002 | 17.2 (16.5-17.9) | 10.8 (10.3-11.2) | 13.4 (13.0-13.8) |
| 2003 | 16.9 (16.2-17.5) | 10.3 (9.8-10.7) | 13.0 (12.7-13.4) |
| 2004 | 15.1 (14.4-15.7) | 10.1 (9.7-10.6) | 12.1 (11.8-12.5) |
| 2005 | 15.1 (14.4-15.7) | 10.0 (9.6-10.4) | 12.1 (11.8-12.5) |
| 2006 | 13.8 (13.2-14.3) | 9.8 (9.4-10.2) | 11.4 (11.0-11.7) |
| 2007 | 13.5 (12.9-14.0) | 8.9 (8.5-9.3) | 10.8 (10.5-11.1) |
| 2008 | 13.4 (12.8-14.0) | 9.2 (8.8-9.6) | 10.9 (10.6-11.2) |
| 2009 | 13.1 (12.6-13.6) | 9.1 (8.7-9.5) | 10.8 (10.5-11.2) |
| 2010 | 13.2 (12.7-13.8) | 9.1 (8.7-9.5) | 10.8 (10.5-11.2) |
| 2011 | 12.7 (12.2-13.2) | 9.3 (8.9-9.7) | 10.7 (10.4-11.1) |
| 2012 | 12.8 (12.3-13.3) | 9.1 (8.7-9.4) | 10.6 (10.3-11.0) |
| 2013 | 12.4 (11.9-12.9) | 8.8 (8.4-9.2) | 10.3 (10.0-10.6) |
| 2014 | 12.6 (12.1-13.1) | 9.2 (8.8-9.5) | 10.7 (10.4-11.0) |
| 2015 | 13.0 (12.5-13.5) | 9.4 (9.0-9.8) | 11.0 (10.7-11.3) |
| 2016 | 13.6 (13.1-14.1) | 10.2 (9.9-10.6) | 11.7 (11.4-12.0) |
| 2017 | 13.9 (13.4-14.4) | 10.8 (10.4-11.2) | 12.1 (11.8-12.4) |
| 2018 | 13.5 (13.0-14.0) | 10.7 (10.3-11.1) | 11.9 (11.6-12.2) |
| 2019 | 13.7 (13.2-14.1) | 10.8 (10.4-11.2) | 12.0 (11.7-12.3) |
| 2020 | 14.1 (13.6-14.6) | 10.9 (10.5-11.3) | 12.3 (12.0-12.6) |
| **Total** | 14.4 (14.3-14.5) | 10.0 (9.9-10.1) | 11.9 (11.8-11.9) |

**Supplemental Table 6:** Lung cancer and secondary brain metastasis–related age-adjusted mortality rates per 100,000, stratified by race, among older adults in the United States, 1999 to 2020.

| **Age-Adjusted Rate (95% CI)** | | | | | |  |
| --- | --- | --- | --- | --- | --- | --- |
| **Year** | **NH White** | **NH Black or African American** | **Hispanic or Latino** | | **NH Asian or Pacific Islander** |  |
| 1999 | 15.9 (15.5-16.4) | 16.6 (15.1-18.1) | 7.5 (6.1-8.8) | | 8.7 (6.8-11.0) |  |
| 2000 | 16.0 (15.5-16.4) | 15.2 (13.8-16.6) | 8.0 (6.6-9.3) | | 7.6 (5.9-9.7) |  |
| 2001 | 15.1 (14.7-15.6) | 14.9 (13.5-16.3) | 5.7 (4.6-6.8) | | 7.3 (5.6-9.3) |  |
| 2002 | 14.2 (13.8-14.6) | 12.1 (10.8-13.4) | 6.5 (5.4-7.7) | | 6.5 (5.0-8.3) |  |
| 2003 | 13.8 (13.4-14.2) | 13.1 (11.8-14.4) | 5.1 (4.2-6.1) | | 7.6 (6.0-9.5) |  |
| 2004 | 12.9 (12.5-13.3) | 11.5 (10.3-12.7) | 6.2 (5.1-7.3) | | 6.6 (5.2-8.4) |  |
| 2005 | 12.9 (12.5-13.3) | 11.7 (10.5-12.9) | 5.7 (4.7-6.7) | | 7.3 (5.8-9.1) |  |
| 2006 | 12.1 (11.7-12.5) | 11.0 (9.8-12.1) | 4.7 (3.8-5.6) | | 5.9 (4.6-7.4) |  |
| 2007 | 11.6 (11.2-12.0) | 10.2 (9.0-11.3) | 5.1 (4.2-6.0) | | 6.2 (4.9-7.8) |  |
| 2008 | 11.6 (11.3-12.0) | 10.7 (9.6-11.8) | 5.4 (4.5-6.3) | | 5.6 (4.3-7.0) |  |
| 2009 | 11.5 (11.1-11.9) | 10.9 (9.8-12.0) | 5.0 (4.1-5.9) | | 6.6 (5.3-8.1) |  |
| 2010 | 11.5 (11.1-11.9) | 11.4 (10.3-12.6) | 5.1 (4.3-6.0) | | 6.2 (5.0-7.6) |  |
| 2011 | 11.5 (11.1-11.8) | 10.1 (9.1-11.2) | 4.6 (3.8-5.4) | | 8.3 (6.9-9.8) |  |
| 2012 | 11.6 (11.2-11.9) | 10.4 (9.3-11.4) | 4.2 (3.5-4.9) | | 5.2 (4.2-6.5) |  |
| 2013 | 11.1 (10.7-11.4) | 10.4 (9.4-11.5) | 4.5 (3.7-5.2) | | 6.4 (5.2-7.6) |  |
| 2014 | 11.4 (11.0-11.8) | 9.9 (8.9-10.9) | 5.2 (4.4-6.0) | | 8.0 (6.7-9.3) |  |
| 2015 | 11.8 (11.4-12.1) | 10.4 (9.4-11.4) | 5.9 (5.1-6.7) | | 7.8 (6.6-9.0) |  |
| 2016 | 12.6 (12.2-12.9) | 11.8 (10.7-12.8) | 5.4 (4.7-6.2) | | 6.9 (5.8-8.1) |  |
| 2017 | 13.1 (12.7-13.4) | 12.2 (11.2-13.2) | 5.4 (4.7-6.1) | | 8.6 (7.4-9.8) |  |
| 2018 | 12.7 (12.4-13.1) | 12.2 (11.2-13.2) | 5.8 (5.1-6.6) | | 7.8 (6.7-8.9) |  |
| 2019 | 12.9 (12.6-13.3) | 12.1 (11.1-13.1) | 5.6 (4.9-6.3) | | 9.6 (8.4-10.8) |  |
| 2020 | 13.4 (13.0-13.7) | 11.3 (10.3-12.2) | 5.9 (5.2-6.6) | | 9.3 (8.2-10.5) |  |
| **Total** | 12.7 (12.6-12.8) | 11.7 (11.4-11.9) | 5.5 (5.3-5.7) | | 7.5 (7.2-7.8) |  |
| NH = non-Hispanic. |  |  |  |  | |  |

**Supplemental Table 7:** Lung cancer and secondary brain metastasis–related age-adjusted mortality rates per 100,000, stratified by states, among older adults in the United States, 1999 to 2020.

| **State** | **Age-Adjusted Rate (95% CI)** | **Rank** | **Percentile** |
| --- | --- | --- | --- |
| Arkansas | 24.5 | 1 | 100 |
| Mississippi | 20.6 | 2 | 98 |
| Vermont | 20.1 | 3 | 96 |
| Indiana | 19.7 | 4 | 94 |
| West Virginia | 16.8 | 5 | 92 |
| Iowa | 16.6 | 6 | 88 |
| Tennessee | 16.6 | 6 | 88 |
| Kentucky | 16.2 | 8 | 86 |
| Delaware | 15.6 | 9 | 84 |
| South Carolina | 15.4 | 10 | 82 |
| Nevada | 15 | 11 | 80 |
| Texas | 14.8 | 12 | 78 |
| Washington | 14.6 | 13 | 76 |
| New Hampshire | 14.4 | 14 | 74 |
| Kansas | 14.3 | 15 | 72 |
| North Dakota | 13.9 | 16 | 70 |
| Ohio | 13.8 | 17 | 68 |
| Nebraska | 13.3 | 18 | 66 |
| South Dakota | 12.3 | 19 | 64 |
| Rhode Island | 12.2 | 20 | 62 |
| Alaska | 12.1 | 21 | 56 |
| North Carolina | 12.1 | 21 | 56 |
| Oregon | 12.1 | 21 | 56 |
| Michigan | 11.9 | 24 | 54 |
| Missouri | 11.7 | 25 | 52 |
| Illinois | 11.6 | 26 | 50 |
| Hawaii | 11.4 | 27 | 46 |
| Maine | 11.4 | 27 | 46 |
| Idaho | 11.1 | 29 | 42 |
| Pennsylvania | 11.1 | 29 | 42 |
| Minnesota | 11 | 31 | 40 |
| California | 10.9 | 32 | 38 |
| Oklahoma | 10.7 | 33 | 36 |
| New York | 10.4 | 34 | 34 |
| Florida | 10.3 | 35 | 32 |
| Connecticut | 9.9 | 36 | 28 |
| Wisconsin | 9.9 | 36 | 28 |
| New Jersey | 9.8 | 38 | 26 |
| Maryland | 9.6 | 39 | 24 |
| Colorado | 9.4 | 40 | 20 |
| Georgia | 9.4 | 40 | 20 |
| Wyoming | 9.2 | 42 | 18 |
| Massachusetts | 8.9 | 43 | 16 |
| Alabama | 8.8 | 44 | 12 |
| New Mexico | 8.8 | 44 | 12 |
| Virginia | 8.7 | 46 | 10 |
| Louisiana | 8.6 | 47 | 8 |
| Montana | 8.2 | 48 | 6 |
| Arizona | 7.7 | 49 | 4 |
| District of Columbia | 7.6 | 50 | 2 |
| Utah | 4.3 | 51 | 0 |

**Supplemental Table 8:** Lung cancer and secondary brain metastasis–related age-adjusted mortality rates per 100,000, stratified by census region, among older adults in the United States, 1999 to 2020.

| **Census Region** | **Year** | **Age-Adjusted Rate (95% CI)** |
| --- | --- | --- |
| Northeast | 1999 | 14.6 (13.7-15.5) |
| Northeast | 2000 | 13.8 (13.0-14.7) |
| Northeast | 2001 | 13.7 (12.8-14.5) |
| Northeast | 2002 | 13.4 (12.6-14.3) |
| Northeast | 2003 | 12.2 (11.4-13.0) |
| Northeast | 2004 | 11.2 (10.4-12.0) |
| Northeast | 2005 | 10.7 (9.9-11.4) |
| Northeast | 2006 | 11.2 (10.4-12.0) |
| Northeast | 2007 | 10.6 (9.9-11.4) |
| Northeast | 2008 | 10.0 (9.2-10.7) |
| Northeast | 2009 | 9.3 (8.6-10.0) |
| Northeast | 2010 | 9.7 (9.0-10.4) |
| Northeast | 2011 | 9.8 (9.1-10.5) |
| Northeast | 2012 | 9.8 (9.210.5) |
| Northeast | 2013 | 9.3 (8.7-10.0) |
| Northeast | 2014 | 8.9 (8.3-9.6) |
| Northeast | 2015 | 8.9 (8.3-9.6) |
| Northeast | 2016 | 10.1 (9.4-10.8) |
| Northeast | 2017 | 9.0 (8.4-9.6) |
| Northeast | 2018 | 9.0 (8.4-9.6) |
| Northeast | 2019 | 8.8 (8.2-9.4) |
| Northeast | 2020 | 10.1 (9.5-10.7) |
| Northeast | **Total** | 10.5 (10.4-10.7) |
| Midwest | 1999 | 16.1 (15.2-16.9) |
| Midwest | 2000 | 16.7 (15.8-17.6) |
| Midwest | 2001 | 16.4 (15.5-17.3) |
| Midwest | 2002 | 14.8 (13.9-15.6) |
| Midwest | 2003 | 14.3 (13.5-15.1) |
| Midwest | 2004 | 13.9 (13.1-14.7) |
| Midwest | 2005 | 12.7 (12.0-13.5) |
| Midwest | 2006 | 12.1 (11.4-12.8) |
| Midwest | 2007 | 12.5 (11.8-13.3) |
| Midwest | 2008 | 11.1 (10.4-11.8) |
| Midwest | 2009 | 11.1 (10.4-11.8) |
| Midwest | 2010 | 11.4 (10.7-12.1) |
| Midwest | 2011 | 11.1 (10.4-11.8) |
| Midwest | 2012 | 10.9 (10.2-11.5) |
| Midwest | 2013 | 11.3 (10.7-12.0) |
| Midwest | 2014 | 12.0 (11.3-12.6) |
| Midwest | 2015 | 12.5 (11.8-13.2) |
| Midwest | 2016 | 13.8 (13.1-14.6) |
| Midwest | 2017 | 13.0 (12.3-13.7) |
| Midwest | 2018 | 13.7 (13.0-14.4) |
| Midwest | 2019 | 14.0 (13.3-14.6) |
| Midwest | 2020 | 13.0 (12.9-13.2) |
| Midwest | **Total** | 16.1 (15.2-16.9) |
| South | 1999 | 16.4 (15.7-17.1) |
| South | 2000 | 16.0 (15.3-16.7) |
| South | 2001 | 15.0 (14.3-15.7) |
| South | 2002 | 13.7 (13.0-14.3) |
| South | 2003 | 13.8 (13.2-14.4) |
| South | 2004 | 12.4 (11.8-13.0) |
| South | 2005 | 12.4 (11.8-12.9) |
| South | 2006 | 11.5 (10.9-12.1) |
| South | 2007 | 10.8 (10.3-11.4) |
| South | 2008 | 11.2 (10.7-11.8) |
| South | 2009 | 11.7 (11.1-12.2) |
| South | 2010 | 11.7 (11.1-12.2) |
| South | 2011 | 11.3 (10.8-11.8) |
| South | 2012 | 11.3 (10.7-11.8) |
| South | 2013 | 11.1 (10.6-11.6) |
| South | 2014 | 11.7 (11.2-12.2) |
| South | 2015 | 11.9 (11.4-12.4) |
| South | 2016 | 12.7 (12.1-13.2) |
| South | 2017 | 13.1 (12.5-13.6) |
| South | 2018 | 12.7 (12.2-13.2) |
| South | 2019 | 13.0 (12.5-13.5) |
| South | 2020 | 12.8 (12.3-13.3) |
| South | **Total** | 12.5 (12.4-12.6) |
| West | 1999 | 13.5 (12.6-14.3) |
| West | 2000 | 13.7 (12.8-14.6) |
| West | 2001 | 11.8 (11.0-12.6) |
| West | 2002 | 11.2 (10.4-12.0) |
| West | 2003 | 11.1 (10.3-11.8) |
| West | 2004 | 10.7 (10.0-11.5) |
| West | 2005 | 11.1 (10.4-11.9) |
| West | 2006 | 9.9 (9.2-10.6) |
| West | 2007 | 9.5 (8.8-10.2) |
| West | 2008 | 9.6 (8.9-10.3) |
| West | 2009 | 10.4 (9.7-11.1) |
| West | 2010 | 10.0 (9.3-10.7) |
| West | 2011 | 9.8 (9.2-10.5) |
| West | 2012 | 9.8 (9.2-10.5) |
| West | 2013 | 9.3 (8.7-9.9) |
| West | 2014 | 9.7 (9.0-10.3) |
| West | 2015 | 10.2 (9.6-10.8) |
| West | 2016 | 10.5 (9.9-11.1) |
| West | 2017 | 11.6 (10.9-12.2) |
| West | 2018 | 11.7 (11.1-12.3) |
| West | 2019 | 11.6 (11.0-12.2) |
| West | 2020 | 11.6 (11.0-12.3) |
| West | **Total** | 10.8 (10.6-10.9) |

**Supplemental Table 9**: Lung cancer and secondary brain metastasis–related age-adjusted mortality rates per 100,000, stratified by urban-rural classification, among older adults in the United States, 1999 to 2020.

| **Age-Adjusted Rate (95% CI)** | | |
| --- | --- | --- |
| **Year** | **Metropolitan** | **Nonmetropolitan** |
| 1999 | 15.1 (14.6-15.5) | 16.6 (15.6-17.6) |
| 2000 | 14.9 (14.4-15.3) | 16.9 (15.9-17.9) |
| 2001 | 13.9 (13.5-14.3) | 16.6 (15.6-17.6) |
| 2002 | 12.9 (12.5-13.3) | 15.6 (14.6-16.5) |
| 2003 | 12.7 (12.3-13.1) | 14.6 (13.7-15.5) |
| 2004 | 11.6 (11.2-12.0) | 14.2 (13.4-15.1) |
| 2005 | 11.7 (11.3-12.1) | 14.1 (13.2-14.9) |
| 2006 | 11.0 (10.6-11.3) | 13.2 (12.4-14.1) |
| 2007 | 10.3 (9.9-10.7) | 13.0 (12.2-13.8) |
| 2008 | 10.3 (10.0-10.7) | 13.5 (12.6-14.3) |
| 2009 | 10.3 (9.9-10.6) | 13.1 (12.3-13.9) |
| 2010 | 10.3 (10.0-10.7) | 12.8 (12.0-13.7) |
| 2011 | 10.1 (9.8-10.4) | 13.4 (12.6-14.3) |
| 2012 | 10.3 (9.9-10.6) | 12.5 (11.7-13.3) |
| 2013 | 9.8 (9.5-10.1) | 12.8 (12.0-13.6) |
| 2014 | 10.1 (9.8-10.5) | 13.0 (12.3-13.8) |
| 2015 | 10.6 (10.3-10.9) | 13.0 (12.2-13.8) |
| 2016 | 11.1 (10.7-11.4) | 14.7 (13.9-15.5) |
| 2017 | 11.6 (11.3-11.9) | 14.8 (14.0-15.6) |
| 2018 | 11.4 (11.1-11.8) | 14.1 (13.3-14.9) |
| 2019 | 11.6 (11.2-11.9) | 14.5 (13.7-15.3) |
| 2020 | 11.7 (11.3-12.0) | 15.5 (14.7-16.3) |
| **Total** | 11.4 (11.3-11.5) | 14.2 (14.0-14.3) |

**Supplemental Table 10:** Fifteen leading underlying causes of death among older adults in the United States, 1999 to 2020, with total deaths and age-adjusted mortality rates per 100,000 population.

| **UCD - 15 Leading Causes of Death** | **Deaths** | **Age Adjusted Rate** |
| --- | --- | --- |
| Diseases of heart (I00-I09, I11, I13, I20-I51) | 11525356 | 1244.4 |
| Malignant neoplasms (C00-C97) | 8883038 | 968.1 |
| Cerebrovascular diseases (I60-I69) | 2746311 | 297.4 |
| Chronic lower respiratory diseases (J40-J47) | 2622109 | 286.5 |
| Alzheimer disease (G30) | 1852432 | 199.0 |
| Diabetes mellitus (E10-E14) | 1209255 | 131.6 |
| Influenza and pneumonia (J09-J18) | 1077571 | 116.1 |
| Accidents (unintentional injuries) (V01-X59, Y85-Y86) | 953643 | 103.0 |
| Nephritis, nephrotic syndrome and nephrosis (N00-N07, N17-N19,N25-N27) | 839349 | 91.1 |
| Septicemia (A40-A41) | 605890 | 65.9 |
| Parkinson disease (G20-G21) | 506561 | 55.7 |
| Essential hypertension and hypertensive renal disease (I10, I12, I15) | 502677 | 54.1 |
| Pneumonitis due to solids and liquids (J69) | 349963 | 37.7 |
| COVID-19 (U07.1) | 282836 | 30.6 |
| Chronic liver disease and cirrhosis (K70, K73-K74) | 268791 | 29.0 |
